# Computational Simulation of LAVA Treatment of Thyroid Eye Disease Predicts Soft Tissue Outcome Comparable to Two-Wall Resection

**DOI:** 10.3390/bioengineering11121181

**Published:** 2024-11-22

**Authors:** Matthias Krause, Evgeny Gladilin

**Affiliations:** 1 Department of Oral and Maxillofacial Surgery, Leipzig University, Liebigstraße 12, 04103 Leipzig, Germany; 2 Applied Bioinformatics, German Cancer Research Center, Berliner Str. 41, 69120 Heidelberg, Germany

**Keywords:** thyroid eye disease (TED), decompression surgery, lateral valgization (LAVA), soft tissue mechanics, computational simulation

## Abstract

Thyroid eye disease (TED) is a common extrathyroidal manifestation of hyperthyroidism, typically associated with Graves’ disease (GD). This condition can cause severe functional limitations as well as significant aesthetic concerns. Treatment for TED patients aims to restore functionality and address aesthetic concerns. Surgical TED treatment is usually performed by orbital wall resection, which effectively decompresses intraorbital tissues and corrects the orbital/ocular disorders. Several different scenarios of surgical TED treatment including one-, two-, and three-wall resections are known. More recently, a new minimally invasive technique, the so-called lateral valgization (LAVA) of the orbital wall, was reported to show promising results comparable to conventional wall resection techniques. Due to the relatively limited data on TED treatment, only a few quantitative investigations of alternative TED surgery scenarios exist. In this feasibility study, we estimate the soft tissue outcome of LAVA treatment using computational simulation. Our experimental results show that the amount of intraorbital tissue released into the extraorbital space by LAVA treatment is comparable with the outcome of two-wall resection. Our computational simulation confirms previously reported isolated clinical findings suggesting that the minimally invasive LAVA approach represents an attractive alternative to conventional wall resection approaches for surgical TED treatment.

## 1. Introduction

Thyroid eye disease (TED), also known as Graves’ ophthalmopathy (GO) is an extrathyroidal, orbital manifestation of hyperthyroidism, typically associated with Graves’ disease (GD). It is a rare disorder [1]. The affected patients may suffer from strong stigmatization due to protruding eyeballs and wide-open lid aperture. In addition to the stigmatization, there are severe limitations of eye function, high sensitivity to light, and double vision, which makes it very difficult for patients to move in everyday life, and even threatens blindness due to optic neuropathy. This can have a serious impact on the lives of the patients involved [2]. The rehabilitation for these patients aims to enhance the functional, aesthetic, and psycho-social implications of this condition [3]. The first steps to addressing ocular pathology during the early, active inflammatory phase of GD include restoration of euthyroid metabolism and elimination of additional risk factors (e.g., smoking). Depending on the strength of the disease, conservative anti-inflammatory and immunosuppressive treatment may be necessary. If these treatments fail, orbital decompression surgery may be indicated to reduce the exophthalmos and its associated consequences. The surgical techniques aim to resect orbital bony walls and optionally also superfluous intraorbital fat, which effectively allows for a decrease in the intraorbital pressure [4].

At present, patients who require surgical treatment prefer to be treated with minimally invasive surgical techniques [5]. Recently, deep lateral decompression has gained popularity due to its effectiveness in achieving satisfactory decompression while minimizing complications, particularly lowering the risk of new-onset diplopia [6]. Lateral decompression of the orbit, first published by Kroenlein and Dollinger, was later popularized by Moran [7,8,9]. However, removal of thin bone may cause medial displacement of the temporalis muscle and close the newly decompressed orbital space. Here, the publications of Tessier and Wolfe described the first methods of lateral orbital wall expansion (French school) that involved a valgus osteotomy [10,11]. This approach stops the expansion of the temporal muscle into the free lateral open-wall area and prevents inadequate decompression. The use and benefits of this procedure vs. conventional wall resection scenarios [12] have been recently described in the surgical literature as lateral valgization of the lateral wall (LAVA) [13].

Accurate estimation of mechanical soft tissue rearrangements due to surgical interventions is known to be a difficult task, which cannot be satisfactorily addressed using simple statistical analysis of previous surgery cases. Computational simulation of individual surgery outcomes requires an accurate 3D reconstruction of the patient’s anatomy and advanced numerical tools for the simulation of non-rigid soft issue behavior in response to surgical interventions (e.g., displacement of bone structures). In a number of early works, the feasibility of biomechanical simulation by prediction of orbital tissue and quantitative assessment of surgery outcome have been demonstrated [14,15]. More recent studies have presented an evaluation of computational models vs. post-surgery data, confirming the remarkable accuracy of physics-based simulations of orbital surgery outcomes [16]. This feasibility study adopts a similar approach to computational simulation of 3D soft tissue mechanics to quantitatively assess the effect of LAVA treatment on orbital tissue outcome, in comparison to a more conventional two-wall resection approach previously investigated in [17,18].

## 2. Materials and Methods

### 2.1. Surgical Procedure

The LAVA procedure is performed via a midline upper-lid and transconjunctival lower-lid approach without canthotomy. Bilateral dissection of the lateral wall follows. Optional orbital wall cutting guides can now be positioned and checked with intraoperative navigation to identify the margins and guide the osteotomy boundaries of the lateral orbital wall. Alternatively, the same procedure can be performed without cutting guides using the margins of the individual patient-specific mini-plates as described below. Osteotomies were performed using a piezosurgical approach (MT 11L-10, MT 11R-10, Mectron Medical, Mectron s.p.a., Carasco, Italy) to facilitate the safe and effective division of bony structures in the deep lateral region of the orbit. Valgization was performed after the completion of the lateral wall osteotomy. To achieve the desired degree of valgization, individual patient-specific mini-plates were placed and fixed with six to eight screws (L1^®^ Midface System 1.5 mm, KLS Martin Group, Tuttlingen, Germany). Wound closure was performed using a multilayer approach with Vicryl 4-0 and 7-0 absorbable sutures.

### 2.2. Study Design

In this feasibility study, computer tomography data were obtained from a 67 y.o. male patient diagnosed with TED at a major university hospital in Germany. The patient had severe thyroid eye disease. His visual acuity was 0.2 in the right eye and 0.6 in the left eye, and his Hertel value was 25–100–25 mm preoperatively. He underwent two-wall orbital resection (lateral and floor walls) for the surgical treatment of TED, which was also computationally analyzed in [18]. Here, we adopt a similar computational approach to estimate the amount of orbital tissue released into the extraobrital space by the alternative lateral valgization (LAVA) treatment.

### 2.3. Three-Dimensional Image Processing and Orbital Model Generation

The overall procedure of CT image processing and 3D model generation is similar to [18]. First, the CT data were segmented into fat, bone, muscle, and bulbus regions followed by the generation of surface and volumetric 3D models of these tissues. Then, the relevant fragments of the orbital wall were annotated according to the LAVA surgery plan. For the simulation of the LAVA scenario, the lateral fragment of the orbital wall was displaced to the new position. The displacement of the lateral wall fragment provided the boundary condition for subsequent simulation of the orbital tissue outcome.

### 2.4. Physics-Based Simulation of Decompression Surgery

For computational simulation of decompression surgery resulting from LAVA treatment, the finite element method (FEM) was used. A detailed description of our FEM approach to the simulation of soft tissue and, in particular, orbital mechanics, can be found elsewhere [18,19]. In brief, an isotropic, homogeneous, piece-wise linear elastic (Hookean) approximation of constitutive soft tissue behavior was used. Accordingly, the mechanical response of soft tissues was computed as the solution to the Lamé–Navier PDE of linear elasticity with respect to tissue displacement (**u**) induced by applied forces (**f**):

∆**u** + (1 − 2*ν*)^−1^ grad div **u** = −2(1 + *ν*)E^−1^ **f**. (1)

The boundary value problem given by Equation (1) and the prescribed boundary conditions were solved numerically using the FEM. The values of two material tissue parameters, Young’s modulus (E) and Poisson’s ratio (*ν*), describing the basic elastic properties of soft tissues (i.e., material stiffness and compressibility), are typically unknown in image-based applications, and often estimated from the reference literature [20]. However, values from the literature can substantially deviate from individual tissue properties. Here, individual material parameters and the effective forces (intraorbital pressure) were estimated iteratively by minimizing the difference between simulated and real post-surgery tissue outcomes, as described in [18].

## 3. Results

**Figure 1:**
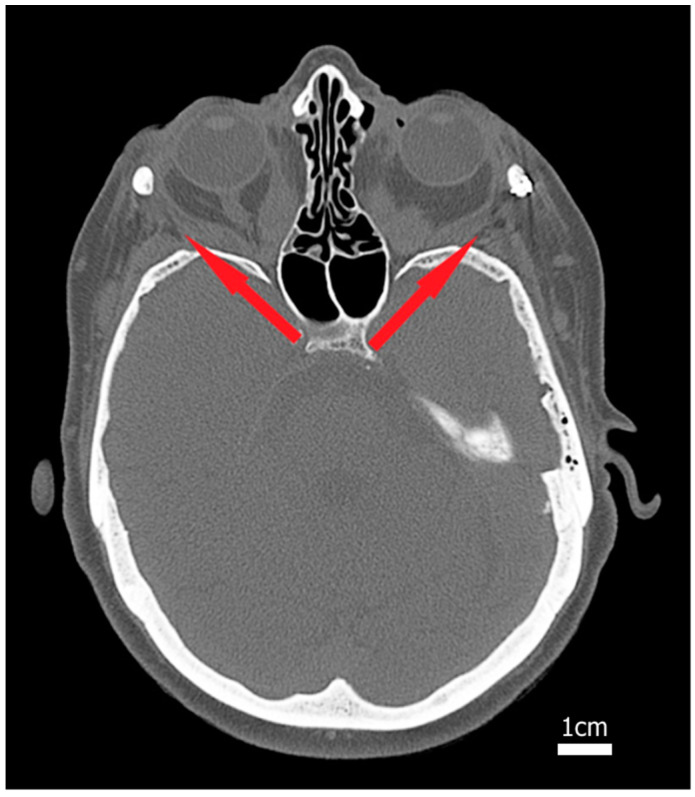
Example of TED treatment using the two-wall resection approach. A central CT slice of a TED patient after two-wall resection treatment (lateral and medial) at both sites. The close relationship of the temporalis muscle to the rectus lateralis muscle can be seen; thus, the laterally open orbit (red arrow) can be closed by the function of the temporalis muscle.

Our clinical observations suggest that the TED treatment strategy using two-wall resection may have a drawback of keeping the position of the temporalis muscle unchanged, which effectively acts as a natural obstacle limiting the outflow of the fat tissue from the orbital space, as shown in Figure 1. The results of previous clinical studies indicate that the soft tissue outcome of LAVA treatment is comparable to two-wall resection; see a summary in Table 1. However, these observations are based on a small number of cases where different patients underwent either one surgical treatment or the other.

**Figure 2:**
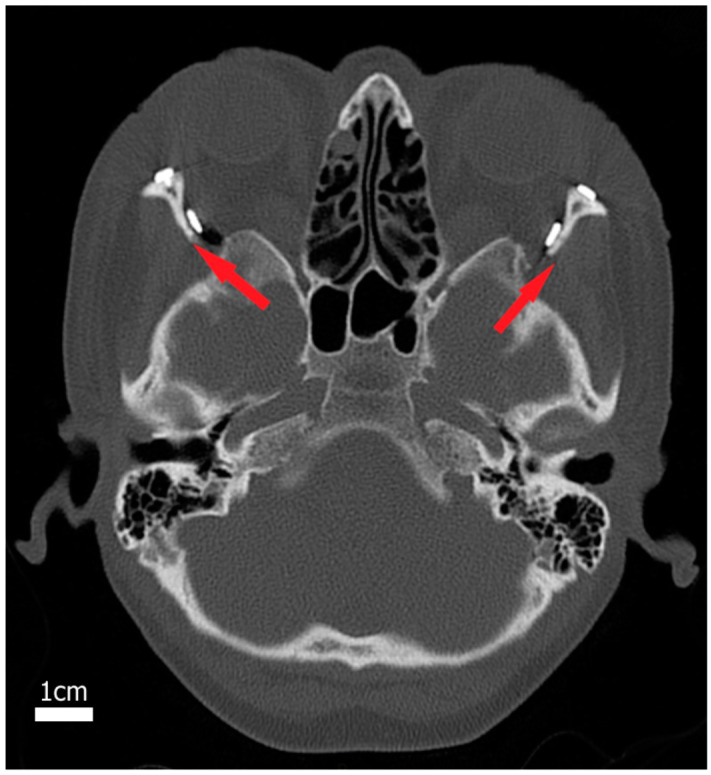
Example of LAVA treatment. A central CT slice of a TED patient who underwent a LAVA treatment. Valgization of the lateral wall at both sites prevents herniation of the temporalis muscle (red arrows) and keeps the new lateral orbital space open.

**Figure 3:**
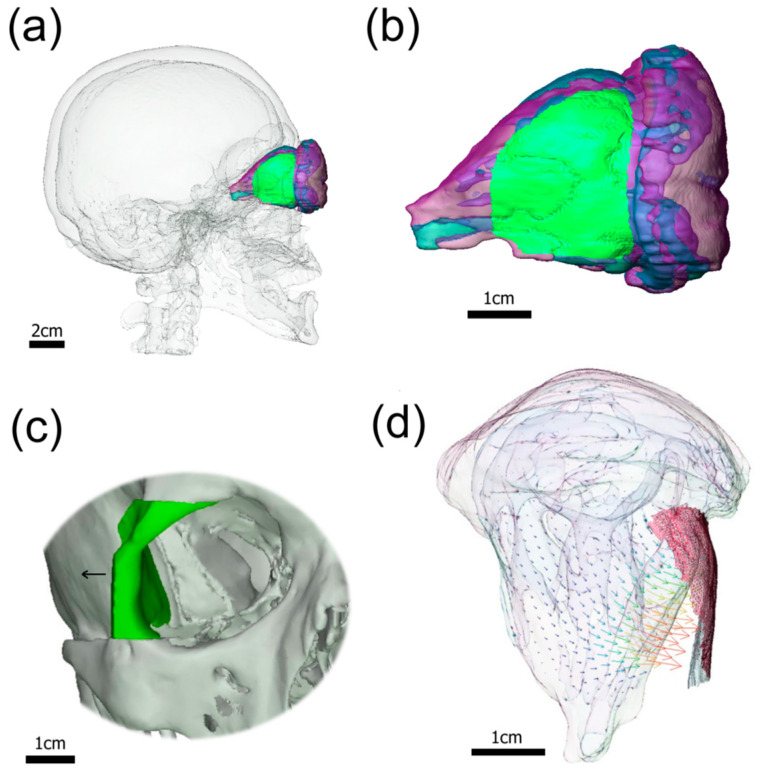
Overview of 3D modeling and simulation of LAVA treatment. (**a**) Side view at the patient skull (transparent) and orbital models (blue–red) including the patch on the right lateral wall (green) to be relocated in the course of the LAVA treatment; (**b**) two-tissue material model of the orbital tissues (red—muscles, blue—fat) with the patch of the lateral wall to be relocated (green); (**c**) front view at the LAVA treatment plan with valgization of the bone fragment of the right lateral wall (green); (**d**) top-view visualization of valgization of the lateral wall followed by an outflow of orbital tissue (indicated by the displacement vectors).

In contrast to wall resection treatment, valgization of the lateral orbital wall in the LAVA approach prevents herniation of the temporalis muscle, which effectively keeps the new lateral orbital space open for outflow of the orbital fat tissue; see Figure 2. To quantitatively assess the impact of surgical treatments on the orbital tissue outcome using LAVA vs. two-wall resection, a computational simulation of 3D orbital mechanics was performed. For a consistent comparison of the simulation results, computational simulation of the LAVA treatment was performed using the same FE orbital model as in our previous study of the two-wall resection [18]. Unlike the two-wall resection scenario, the simulation of LAVA treatment consists of a single valgization affecting only the lateral fragment of the orbital wall. Figure 3a,b show the 3D patient skull and orbital models including the displaced fragment of the orbital wall, which was laterally shifted by 2.5 mm and simultaneously rotated by 30°. Figure 3c visualizes the lateral fragment of the orbital wall, which was virtually resected and relocated to define the boundary conditions for the subsequent FE simulation of the soft tissue outcome. The FE simulation was performed using otherwise the same hyperelastic material model with empirically estimated constitutive parameters (i.e., the relative elastic modulus and compressibility) and forces (intraorbital pressure) as described in [18]. Unlike the two-wall resection scenario, which essentially consists of the removal/opening of lateral and floor wall fragments followed by the outflow of the fat tissue under the impact of uniform intraorbital pressure, the LAVA treatment was simulated in two steps including (i) valgization (i.e., displacement) of the lateral orbital wall affecting the geometry of fat and muscle tissues followed by (ii) outflow of the fat tissue through the open window in the orbital under the impact of the intraorbital pressure. Consequently, the major difference between the two-wall and LAVA scenarios is in the active relocation of orbital muscles, which provides more space for releasing the fat tissue. Our computational simulations showed that the soft tissue outcome of the LAVA scenario, including valgization of a 9.6 cm^2^ segment of the lateral orbital wall, amounted to 7.34 cm^3^ of released intraorbital tissue. This outcome is comparable to the amount of tissue released by the two-wall resection scenario. However, unlike the two-wall resection scenario, a similar amount of tissue outflow was achieved in the LAVA scenario with only half the resection area. Table 2 summarizes the results of the simulated soft tissue outcome for LAVA and two-wall resection scenarios.

## 4. Discussion

It is well known that exophthalmic protrusion is an important, stigmatizing symptom of thyroid eye disease [21]. Depending on the individual circumstances and the strength of the disease, conservative anti-inflammatory and immunosuppressive therapy may be the first line of treatment. If these pharmacological treatments fail, orbital decompression surgery techniques may be indicated to decrease the orbital pressure resulting from the increase in orbital soft tissue volume. The decompression of the lateral orbital wall is popular because it promises minimal complications and is perhaps the first choice for decompressing the orbital cavity [22]. This procedure involves the wide expansion of lateral wall resection to create space for orbital expansion [23]. Despite reports of lower rates of new-onset diplopia with this procedure, lateral wall removal can lead to new problems, including temporal recession, hollowing, and masticatory oscillopsia, especially in procedures that include the resection and removal of the lateral orbital rim [11,22,24]. Unfortunately, in many patients with TED, we see an unsightly lateral retraction preoperatively. Therefore, we prefer to leave the rim intact, in accordance with the findings of previous publications [25]. Another problem associated with these resection techniques of lateral orbit is the herniation of the temporalis muscle into the free orbital space, which may inhibit the outflow of orbital tissue [26]. This negative effect can be prevented by a valgus osteotomy of the intact, lateral wall performed during LAVA [10,11]. This principle has been improved by others, as described in several recent reports [13,25,26]. Compared to the results of our previous biometric analyses, LAVA resulted in effective orbital decompression due to greater lateral wall expansion, which prevents hernitation of the temporalis muscle. [13,17]. As an effect of the lateral wall extension, the intraorbital volume is effectively expanded, especially in the apex region, which also leads to a greater reduction in proptosis [18]. Our computational simulations confirm that a disproportionally large amount of released orbital fat tissue per area of the affected orbital wall can be expected in the case of LAVA treatment compared to two-wall treatment scenarios [12,13]. This confirms our assumption that the LAVA approach effectively preserves more orbital space for releasing superfluous fat tissue by relocating the temporalis muscle, compared to two-wall resection, which leaves the orbital muscles in the same location, limiting the fat tissue outflow. However, we see limitations to this approach. Severe cases of thyroid eye disease with large exophthalmos requiring reduction in proptosis > 8 mm cannot be satisfactorily treated with two-wall resection or LAVA alone. In these cases, the medial orbital wall must also be resected. Digital planning is very time-consuming. There are increasing costs due to the use of dynamic navigation systems and titanium printers for patient-specific implants (PSIs).

## 5. Conclusions

Previous clinical studies have shown the beneficial effects of lateral valgization in the surgical rehabilitation of patients with thyroid eye disease. Our computational feasibility study confirms these observations demonstrating that lateral valgization leads to orbital decompression comparable to more invasive two-wall resection techniques. In conclusion, LAVA valgization seems to be a promising approach in the surgical orbital rehabilitation of patients with TED. Further data on LAVA post-surgery outcome including long-term effects are required to confirm the preliminary results of this feasibility study.

## Figures and Tables

**Table 1:** Summary of clinically observed soft tissue outcome for wall resection vs. LAVA treatment (from [12,13]).

Treatment Scenario	Number of Patients	Resected Area (Average), cm^2^	Post-Surgery Change in the Orbital Volume (Average), cm^3^
One-wall	5	4.97	3.77
Two-wall	94	12.73	5.3
Three-wall	29	18.85	8.6
LARA	99	13.77	5.95
LAVA	18	12.1 *	6.2

*: total valgization area including valgized and new free areas (no resection in the case of LAVA).

**Table 2:** Summary of simulated soft tissue outcome for LAVA treatment vs. two-wall resection (from [18]).

Simulation Scenario	Fragment of the Orbital Wall	Simulated Resection Area, cm^2^	Simulated Change in the Orbital Volume, cm^3^
Two-wall	Lateral (L) area	9.28	1.81
Two-wall	Floor (F) area	9.69	6.08
Two-wall	L + F areas	18.92	7.58
LAVA	Valgization area	9.6 *	7.34

*: simulated valgization area including valgized and new free areas (no resection).

## Data Availability

Patient’s CT data presented in this article are not readily available as public domain for ethical reasons.
